# Prevalence and Antibiotic Resistance Profile of Bacterial Pathogens in Aerobic Vaginitis: A Retrospective Study in Italy

**DOI:** 10.3390/antibiotics10091133

**Published:** 2021-09-20

**Authors:** Enrica Serretiello, Biagio Santella, Veronica Folliero, Domenico Iervolino, Emanuela Santoro, Roberta Manente, Federica Dell’Annunziata, Rossella Sperlongano, Valeria Crudele, Anna De Filippis, Massimiliano Galdiero, Gianluigi Franci, Giovanni Boccia

**Affiliations:** 1 Section of Microbiology and Virology, University Hospital “Luigi Vanvitelli”, 80138 Naples, Italy; enrica.serretiello@unicampania.it (E.S.); bi.santella@gmail.com (B.S.); roberta.manente@studenti.unicampania.it (R.M.); valeria.crudele@unicampania.it (V.C.); 2 Department of Experimental Medicine, University of Campania “Luigi Vanvitelli”, 80138 Naples, Italy; veronica.folliero@unicampania.it (V.F.); federica.dellannunziata@unicampania.it (F.D.); rossella.sperlongano@unicampania.it (R.S.); anna.defilippis@unicampania.it (A.D.F.); massimiliano.galdiero@unicampania.it (M.G.); 3 Department of Public Health and Infectious Diseases, Sapienza University of Rome, 00185 Rome, Italy; iervolino.1886704@studenti.uniroma1.it; 4 Department of Medicine, Surgery and Dentistry “Scuola Medica Salernitana”, University of Salerno, 84081 Baronissi, Italy; esantoro@unisa.it; 5 Dai Dipartimento di Igiene Sanitaria e Medicina Valutativa U.O.C. Patologia Clinica E Microbiologica, Azienda Ospedaliero-Universitaria S. Giovanni di Dio e Ruggi D’Aragona Scuola Medica Salernitana, Largo Cittaà di Ippocrate, 84131 Salerno, Italy

**Keywords:** aerobic vaginitis, antibiotic treatment, multi-drug resistance, antimicrobial resistance

## Abstract

Aerobic vaginitis (AV) is a vaginal infectious condition, characterized by a high inflammatory response and/or signs of epithelial atrophy, a decrease in the amount of *Lactobacillus* spp. and an increase in enteric origin bacteria. AV, often misdiagnosed, is difficult to treat due to the emerging spread of multi-drug resistant bacterial strains. The present study aimed to define the prevalence of AV, to detect causative bacteria and their antimicrobial resistance pattern. Women 10–95 years old, admitted to San Giovanni di Dio e Ruggi d’Aragona Hospital, Salerno, Italy (in the years 2015–2019) are included in the study. Bacterial identification and antibiotic susceptibility tests were carried out by VITEK^®^ 2. Among 2069 patients, 1176 tested positive for microbial growth. A higher incidence of infection was found in the 55–64 age group. Among the pathogenic strains, 50.4% were Gram-negative, and 49.6% were Gram-positive. *Escherichia coli* (*E. coli*) (32.5%) was the most representative strain, followed by *Enterococcus faecalis* (*E. faecalis*) (29.4%), *Klebsiella pneumoniae* (*K. pneumoniae*) (7.8%) and *Enterococcus faecium* (*E. faecium*) (7.7%). *E. coli* showed high sensitivity to carbapenems and amikacin. *K. pneumoniae* carbapenems resistance was fluctuating over time. Alarming resistance to vancomycin was not recorded for *Enterococci*. Both strains were sensitive to teicoplanin, linezolid and tigecycline. Proper diagnosis and an effective therapeutic approach are needed to improve AV management.

## 1. Introduction

The vaginal microbiota is a complex ecosystem, which forms a mutually beneficial relationship with their host and has a significant impact on women’s health. *Lactobacillus* spp. constitute the predominant bacterial population (about 80%) in the vaginal cavity, notably *Lactobacillus crispatus*, *Lactobacillus iners*, *Lactobacillus gasseri* and *Lactobacillus jensenii* [1,2]. *Lactobacillus* spp. cope with the onset of infections through the production of lactic acid, hydrogen peroxide and bacteriocins. Lactic acid represents one of the main products of the fermentation processes of glucose. It confers an acid pH around 3.8–4.5 on the vaginal region, inhibiting the proliferation of pathogenic bacterial species. H_2_O_2_ production by *Lactobacillus* spp. represents an important defense mechanism against pathogenic colonization. Bacteriocins produced by *Lactobacillus* spp. are antimicrobial peptides that are toxic to pathogenic bacteria but pose no threat to the healthy vaginal microbiota [3]. A vaginal bacterial flora imbalance can result in infections such as vulvovaginal candidiasis (CVV), bacterial vaginosis (BV) or aerobic vaginitis (AV). These infectious conditions can cause preterm birth, amniotic fluid infection, chorioamnionitis, sexually transmitted infections and cervical intraepithelial neoplasia [4]. AV is a vaginal infectious condition, which occurs by purulent secretions (fishy odor test negative), meaning inflammation and vaginal epithelial damage. The main AV causative agents are bacteria of intestinal origin. The most frequently isolated bacteria in AV patients are *E. coli*, *K. pneumoniae*, *Enterococcus* spp., *Staphylococcus aureus* (*S. aureus*), Coagulase Negative Staphylococci (CoNS) and *Streptococcus agalactiae* (*S. agalactiae*) [5,6,7]. The AV diagnosis is based on the analysis of the patient’s clinical signs and laboratory tests. AV symptoms are characterized by introital and vaginal redness, yellow discharge, foul odor, redness, itching, burning and dyspareunia. The identification of AV is performed through microscopic analysis, cultural examination and antibiotic susceptibility test. Microscopic analysis of vaginal smears shows *Lactobacillus* spp. deficiency, high load of Gram-positive and/or Gram-negative bacteria and parabasal epithelial cells and/or vaginal leukocytes. An accurate microbiological diagnosis allows the antibiotic sensitivity profiles of aerobic vaginal pathogens to be identified for rapid recovery and prevention of complications. Several studies report that the incidence of aerobic vaginitis ranges from 5 to 80%, widely variable as determined by socio-demographic factors, such as ethnicity, geographic location and socio-economic status [8]. The worldwide problem of aerobic vaginitis is strongly linked to the emergence of antimicrobial resistance (AMR). Women with AV can develop a negative outcome following the failure of antibiotic treatment [9]. The high incidence of AV and drug-resistant bacterial strains highlights the need for better knowledge of AV-causing microorganisms and their antibiotic resistance profiles. The purpose of this retrospective study was to define the most prevalent AV causative aerobic bacteria and their antimicrobial susceptibility profiles isolated in women admitted at San Giovanni di Dio e Ruggi d’Aaragona Hospital, Italy, from 2015 to 2019.

## 2. Results

### 2.1. Incidence of AV Positive and Age Patient Distribution

During the period from 1 January 2015 to December 2019, 2069 vaginal swabs were collected at the San Giovanni di Dio and Ruggi d’Aragona Hospital, Salerno, Italy. Of these, 1176 tested positive for microbial growth and processed for subsequent identification (Table 1).

The investigation was performed on vaginal swabs collected by women aged between 10 and 95 years. The distribution of positive samples by age group is shown in Table 2. The highest incidence of positive results was found in age group 55–64 (26.5%), followed by 35–44 (17.9%), 25–34 (15.2%), 65–80 (15%), 45–54 (14.1%), 15–24 (7.2%), 81–95 (3.4%) and in women under the age of 14 (0.6%).

### 2.2. Prevalence of Isolated Bacteria 

**Figure 1:**
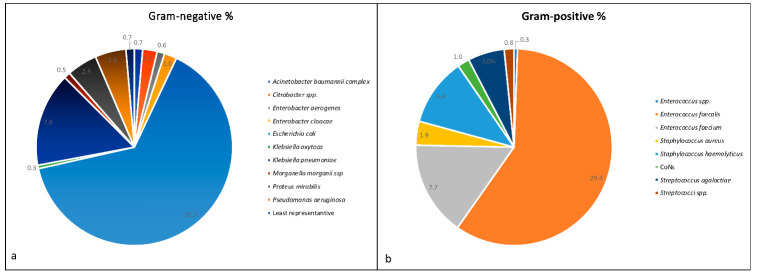
Percentage of (**a**) Gram-negative and (**b**) Gram-positive bacteria found in positive vaginal swabs. In (**a**) “least representative” signifies strains isolated only one time during the five years, so these strains were merged together into one category. In (**b**) CoNs represents coagulase-negative staphylococci.

Among the 50.4% Gram-negative bacteria isolated, *E. coli* showed the highest incidence of 32.5%, followed by *K. pneumoniae* (7.8%) (Figure 1a). Among the 49.6% Gram-positive isolated bacteria, the most represented was *E. faecalis* with an incidence of 29.4%, followed by *E. faecium* (7.7%) (Figure 1b).

**Figure 2:**
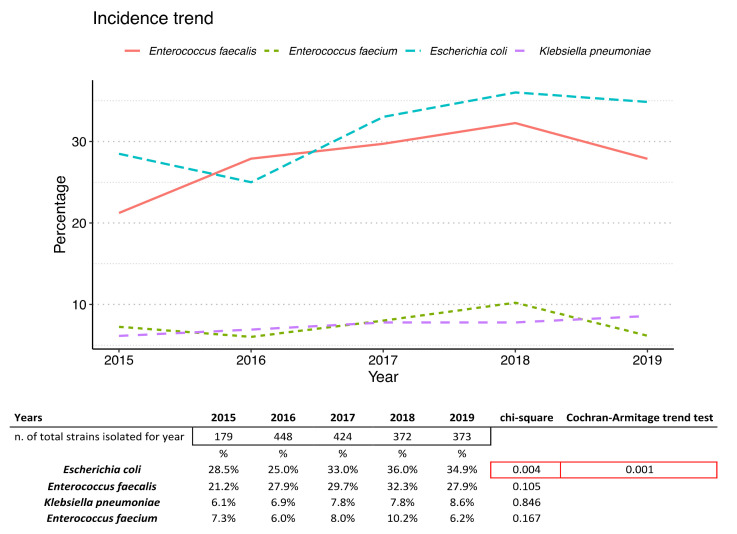
Incidence trend of the main representative bacteria isolated during the 5 years investigated.

The incidence trend of the most representative bacteria during the years is reported in Figure 2.

The incidence of the most abundant bacteria shows an almost constant trend over time except for *E. coli*. A relationship between incidence increase and time was found (chi square *p*-value = 0.004), and an increasing trend was statistically significant (Cocrhran–Armitage trend test = 0.001).

### 2.3. Antimicrobial Susceptibility Pattern

**Figure 3:**
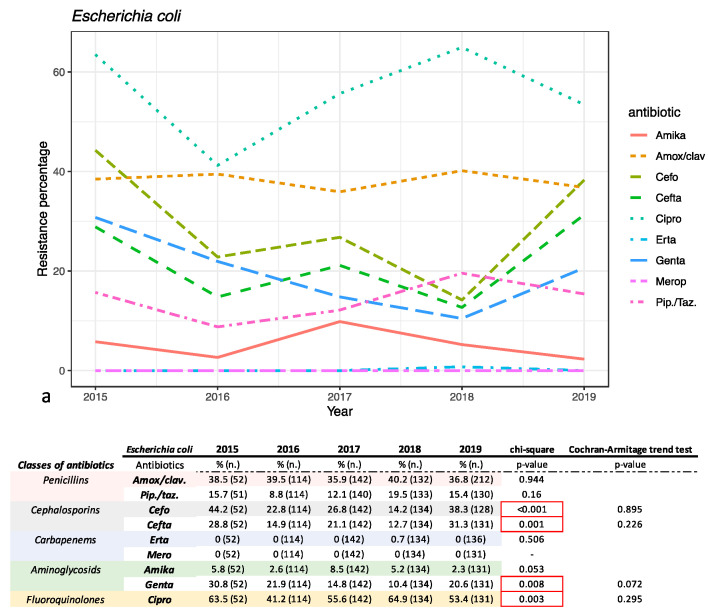

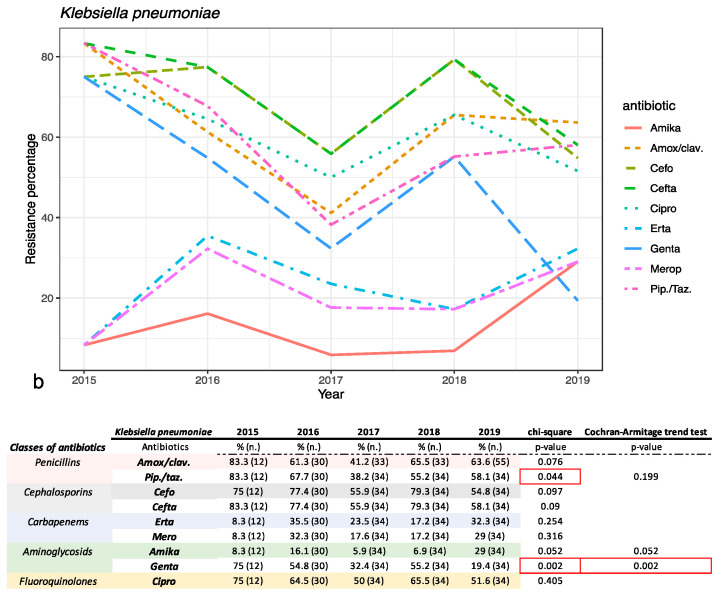
(**a**) *E. coli* and (**b**) *K. pneumoniae* antimicrobial susceptibility trend percentage is reported for each year investigated in the study. The table under the image reports the relative resistance percentage analyzed in the chart, divided by classes of antibiotics tested. % = % of resistance for each year; *n*. = number of total assays conducted for each year. Abbreviations: Amox./clav. = amoxicillin/clavulanic acid; Pip./Taz. = Piperacillin/tazobactam; Cefo. = Cefotaxime; Cefta. = Ceftazidime; Erta. = Ertapenem; Merop. = Meropenem; Amika. = amikacina; Genta. = Gentamicin; Cipro.= ciprofloxacin.

**Figure 4:**
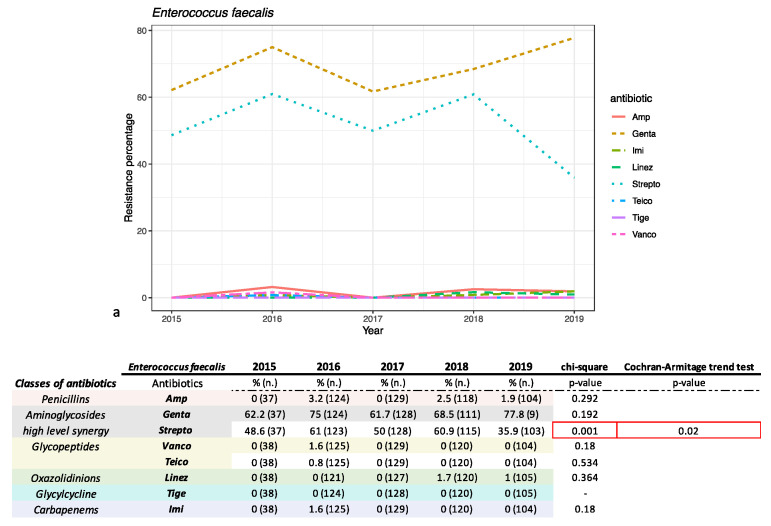

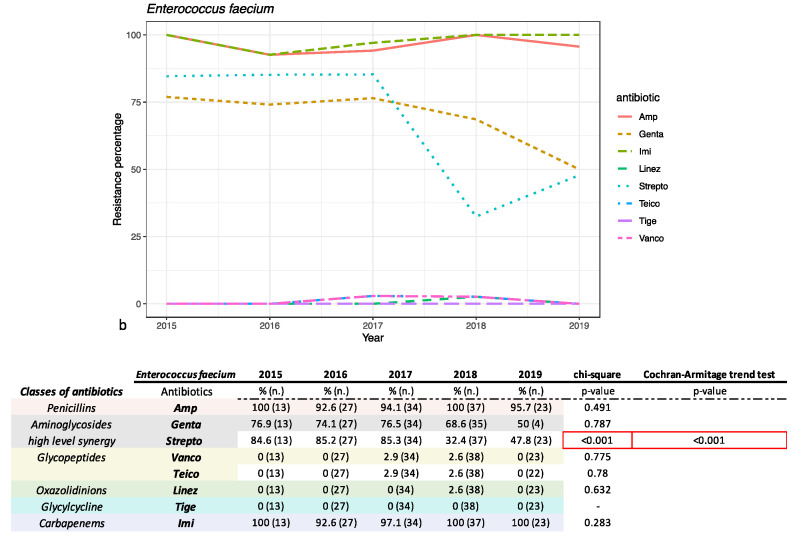
(**a**) *E. faecium* and (**b**) *E. faecalis* antimicrobial susceptibility trend percentage is reported for each year investigated in the study. The table under the image reports the relative resistance percentage analyzed in the chart, divided by classes of antibiotics tested. % = % of resistance for each year; *n*. = number of total assays conducted for each year. Abbreviations: Amp = ampicillin; Genta = Gentamicin; Strepto = streptomycin; Vanco = Vancomycin; Teico = teicoplanin; Linez = Linezoli; Tige = Tigecycline; Imi = Imipenem.

The antimicrobial susceptibility pattern of the most representative Gram-negative and Gram-positive bacteria was investigated and is reported in Figure 3 and Figure 4, respectively. 

**Figure 5:**
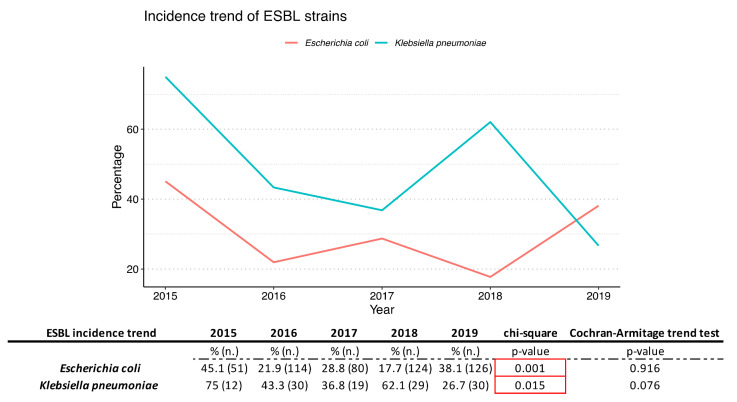
Incidence trend of *E. coli* producing ESBL and *K. pneumoniae* producing ESBL. %= % of resistance for each year; *n*. = number of total assays conducted for each year.

Data reported and discussed below refer to the medium value from the five years analyzed. For *E. coli* a low rate of resistance was recorded for carbapenems (0.17%) and amikacin (4.89%). Resistance rates below 27.2% were found for piperacillin/tazobactam (14.26%), gentamicin (17.98%), ceftazidime (20.9%) and cefotaxime (27.2%). For gentamicin, ceftazidime, cefotaxime and ciprofloxacin (55.1% resistance) a relationship between incidence and time was found (chi-square *p*-value < 0.05), even if there was not a statistically relevant trend (Cochran–Armitage trend test > 0.05). Resistance of 37.88% was shown for amoxicillin/clavulanic acid (chi-square *p*-value > 0.05) (Figure 5). *K. pneumoniae* was more resistant than *E. coli*. Indeed, these strains showed higher resistance to meropenem (22.6%), ertapenem (25.5%) and amikacin (13.9%) compared to *E. coli*. The percentages of resistance ranged from 30 to 67% for ceftazidime (68.6%), gentamicin (43%), piperacillin/tazobactam (56.9%), amoxicillin/clavulanic acid (60.2%), ciprofloxacin (59.1%) and cefotaxime (67.1%) (Figure 5). In contrast, gentamicin resistance showed a decreasing trend in the five years analyzed (chi-square and Armitage trend tests both with *p*-value = 0.02).

Our study also evaluated the prevalence of *E. coli* and *K. pneumoniae* producing extended spectrum beta lactamases (ESBLs) in period study (Figure 5). Both strains showed a variation of incidence statistically relevant during the years investigated (chi-square *p*-value < 0.05).

Among Gram-positive bacteria, *E. faecalis* was found to be more sensitive than *E. faecium* (Figure 4a,b) as expected. No resistance to tigecycline was shown for *E. faecalis*. A resistance rate lower than 1.76% was recorded for vancomycin and teicoplanin (0.4% and 0.2%), linezolid (0.6%), imipenem (0.8%) and ampicillin (1.7%). Resistance rates higher than 52.2% were found for aminoglycosides (gentamicin 68%, streptomycin 52.2%). In particular, streptomycin showed a decreasing, statistically relevant, trend (chi-square *p*-value = 0.01 and Armitage trend test *p*-value = 0.02). *E. faecium* showed a different resistance profile compared to *E. faecalis*: 98% and 96.3% for imipenem and ampicillin, respectively. Both *Enterococci* shared resistance to gentamicin (72.6%) and streptomycin (64.18%). As with *E. faecalis*, *E. faecium* showed a decreasing trend for streptomycin resistance (both chi-square *p*-value and Armitage trend test with *p*-value < 0.01). It showed high sensitivity to tigecycline (0.00% of resistance), vancomycin and teicoplanin (1.5% and 1.5%) and linezolid (0.7%).

## 3. Discussion

In 2002 Donders et al. defined with the term AVs a type of abnormal vaginal flora, distinct from bacterial vaginosis for etiology and pathogenesis. Successfully, in 2011 they correlated AVs in pregnant women to the risk of a negative pregnancy such as preterm birth, premature rupture of membranes, and negative results on the newborn, fetal infections and neurological injury [10,11]. Complications of AV not properly diagnosed or adequately treated in non-pregnant women have not yet been sufficiently investigated, but treatment for AV is strongly suggested to be based on antibiotic susceptibility models [12]. Monitoring the incidence and epidemiology of this infection is a useful strategy to prevent the increase in the risk of maternal and neonatal morbidity. The present study reports the incidence of Gram-negative and Gram-positive bacteria isolated from vaginal swabs of women admitted at San Giovanni di Dio e Ruggi d’Aragona Hospital, Salerno, Italy. Several papers investigated AV in women in a single range of age, such as the reproductive years (about 20–40). Our study investigated women from 10 to 95 years, showing that the greater incidence of AV is recorded in women aged 55–64 years. The explanation could lie either in a correlation with hormonal phases or in recurrent AV episodes due to previous therapeutic failures. Alternatively, a decrease in *Lactobacillus* spp. is correlated with increasing age [13].

From our data it emerged that the most abundant isolated strain was *E. coli* (32.5%), followed by *E. faecalis* (29.4%), *K. pneumoniae* (7.8%) and *E. faecium* (7.7%), and their incidence trend appears constant over the years. The variation in *E. coli* over time shows a statistically significant increasing trend (*p*-value = 0.01), while the variations over the years of the other pathogens analyzed do not seem to be significant. Although the percentage of incidence of pathogenic bacteria changed in accordance with the local epidemiology and the type of population examined in the different studies (age group, etiology and demographic conditions), the obtained data are in line with the literature for the same context [14,15,16]. Sangeetha K. T. et al. in 2015 observed that the most common aerobic bacterial pathogens associated with vaginitis isolated from women patients of reproductive age were *E. faecalis* (32.26%), followed by *E. coli* (25.8%), *S. aureus* (19.35%) and β-hemolytic streptococci (9.68%) [7]. In contrast, in a recent paper Vidyasagar V. (2021) identified, in women diagnosed with aerobic vaginitis (29–35 years), coagulase-negative Staphylococci growth in 50% cases, and in 25% Streptococci and Klebsiella [8]. *S. aureus* was the most abundant isolated pathogen for Nahar D et al. (41.07%), Tansarli et al. (41.7%) and Zarbo et al. (27.9%) [12,17,18]. 

The sensitivity to different categories of antibiotics and their variation over time was analyzed and reported for *E. coli, K. pneumoniae, E. faecium* and *E. faecalis. E. coli* was found to be very sensitive to carbapenems and amikacin, whereas it was found to be moderately sensitive to piperacillin/tazobactam, gentamicin, and third-generation cephalosporins. In particular, for ceftazidime, cefotaxime, ciprofloxacin and gentamicin, an association between the years analyzed and resistance to the antibiotic was found, albeit without showing a real trend over time (chi square *p*-value < 0.05). The resistance percentage of more than 20% for cefotaxime and ceftazidime is an alarming figure. The resistance to ciprofloxacin was high for *E. coli*. For *K. pneumoniae*, resistance to penicillin amoxicillin/clavulanic acid and piperacillin/tazobactam saw a decrease during the five years analyzed, but only for piperacillin/tazobactam was is statistically relevant (chi square *p*-value < 0.05). The cephalosporins cefotaxime and ceftazidime did not show a statistically significant trend (for both, chi-square *p*-value > 0.05). The trend of resistance to carbapenems has been fluctuating over the years (chi square *p*-value > 0.05) and must certainly be monitored in the future. An important decrease occurred for resistance to gentamicin and ciprofloxacin, although only the former was statistically significant (chi-square *p*-value < 0.05). In fact, this significant decrease in resistance is only seen with gentamicin. Strains of *E. coli* and *K. pneumoniae* producing extended spectrum beta lactamases (ESBLs) have been highlighted. The incidence of positive ESBLs has fluctuated greatly over 5 years and, although there was no real trend, the incidence of ESBLs showed a statistically significant variation over time (both *p*-values > 0.05).

Both enterococci exhibited the well-known resistance to the aminoglycosides, even if used as high-level synergy: 72.6% resistance for gentamicin and 64.2% for streptomycin for *E. faecium*, and 68% and 52.2% for *E. faecalis*, even as both strains showed a decrease during the period analyzed (*p*-value < 0.05). Whereas *E. faecalis* resulted in high sensitivity to ampicillin (1.8%) and imipenem (0.8%), *E. faecium* showed high resistance for both, 96.3% and 98%, respectively. The encouraging fact is that resistance to vancomycin has undergone variations over time but ended up decreasing. Tigecycline and linezolid are efficient at 100% for both cocci. *E. faecium* were more resistant compared to *E. faecalis*. The data reported in this work are in line with other retrospective and non-retrospective studies [7,12,19,20,21]. Furthermore, the percentages of resistance for the most worrying pathogens identified in the paper (*E.coli, K. pneumoniae, E. faecium* and *E. faecalis*) are in line with the data collected in Italy and exposed in the AR-ISS 2019 report for most of the molecules tested in the study [22].

Since AVs are characterized by a different clinical condition than BVs (a non-inflammatory vaginal bacterial infectious condition), they need not only to be immediately distinguished from them, but also to be adequately managed. In fact, antibiotic treatment may not be sufficient for most AV patients due to the amount of inflammation associated with this condition. Although there is no generally accepted clinical strategy for the treatment of AV, a combined treatment of antibiotics useful for the treatment of the infectious agent, topical steroids aimed at reducing the inflammatory state and estrogens to treat atrophy has been proposed [14]. Obviously, the treatment must be framed according to local development conditions, especially for rural areas in the developed countries. In general, AV does not respond well to metronidazole treatment, which is more effective on BV. Clindamycin remains a good option, even for pregnant women with severe flora imbalances, as well as fluoroquinolones and topical use of kanamycin, accompanied by local vaginal administration of *lactobacilli* [23,24,25]. Systemic therapy with moxifloxacin may be suitable, especially in the treatment of *S. aureus* resistant to methicillin [15]. Results from our data showed that the infections mediated by *E. coli* could be managed with different classes of antibiotics because it is sensitive to cephalosporins, aminoglycosides and carbapenems. For *K. pneumoniae*, a resistant strain, an approach with aminoglycoside, carbapenems and tigecycline is suggested. Enterococci showed little or no resistance to molecules such as linezolid, vancomycin, teicoplanin or tigecycline. However, it is necessary to emphasize that the use of carbapenems in general and of vancomycin must be limited in order to limit antibiotic resistance spread. Moreover, it is important to monitor the maternal colonization status of ESBL-producing bacteria. The presence of ESBL strains is a risk factor for transmission to the newborn and is associated with preterm birth and premature rupture of membranes (pPROM), which shows the need to revisit diagnostic guidelines and implement therapeutic approaches [26,27,28]. In addition to the very important aspect related to pregnancy birth rate, it is important to collect information on AV pathogens and their resistance profiles to prevent infections in women of childbearing age and safeguard them from the outcome of an AV pregnancy. 

## 4. Materials and Methods

### 4.1. Samples Collection 

In the period from January 2015 to December 2019, a total of 2069 vaginal swabs were collected from patients admitted to the University Hospital “San Giovanni di Dio e Ruggi d’Aragona”, Salerno, Italy, and processed in a microbiological laboratory as described below.

### 4.2. Inclusion and Exclusion Criteria

The samples tested met the following inclusion criteria: (i) patients aged 10 to 95; (ii) female patients hospitalized were included in the analysis; (iii) presence of leucocytes (>25% for each field observed); (iv) decrease or absence of *Lactobacilli* spp.; (v) presence of parabasal epithelial cells. Exclusion criteria were: (i) presence of commensal bacterial growth; (ii) women under the age of 10 were not included in the analysis; (iii) women who have taken antibiotics up to seven days prior the analysis, or who have used chemical cleansers for vaginal douches, were not included. 

### 4.3. Bacterial Culture 

The samples were sown on BBL Trypticase Soy Agar with 5% Sheep Blood, MacConkey II Agar, CNA agar + 5% sheep blood, Chocolate agar PolyViteX, Sabouraud Glucose Agar (Becton Dickinson, NJ, USA) and incubated for 24 h at 37 °C (48 h in a 5% CO_2_ atmosphere for Chocolate agar). Samples with a positive number for pathogenic bacterial growth were processed for the bacterial identification and antimicrobial sensitivity test. 

### 4.4. Bacterial Identification and Antibiotic Susceptibility Test

After each plate examination, bacterial identification and antimicrobial susceptibility tests were performed via technology VITEK^®^2 (BioMe’rieux, France), following the manufacturer’s recommendations. The results of antimicrobial susceptibility were interpreted as “susceptible”, “resistant” or “intermediate” according to EUCAST guidelines and obtained after 16 h of incubation. The antimicrobials molecules were tested in accordance with the typology of bacteria analyzed, following the manufacturer’s recommendations.

### 4.5. Data Analysis

Microsoft Excel 2019 (Microsoft Corp.) was used as data analysis software to elaborate patient’s demographic data (age, sex), number of strains isolated and their antimicrobial pattern. Chi-square tests were used to verify the existence of a possible association between the strain’s incidence or the variation in antibiotic resistance, and the variable time. When an association was found, we proceeded with the Cochran–Armitage trend test to verify the hypothesis of the existence of a trend. For both tests a confidence value of alpha equal to 5% was evaluated. Data were interpreted on the *p*-value score. A *p*-value > 0.05 showed a non-association between variation in incidence of a pathogen/variation in resistance as a function over time; therefore, they are not commented on in the text. Being statistically insignificant, the Cochran–Armitage trend test was not applied. In the case of chi square *p*-value < 0.05, the association was verified; therefore, the Cochran–Armitage trend test was applied: a *p*-value < 0.05 confirms the existence of a trend. IBM Statistical Package for Social Sciences Version 22.00 (IBM SPSS Inc., Armonk, NY, USA) was used to perform the statistical analyses [29].

#### 4.5.1. Ethical Consideration Statement

Our retrospective study is based on laboratory management data collected from databases and is not directly associated with patients. For this reason, ethics approval from the Human Research Ethics Committee was not required for this study. 

#### 4.5.2. Limitations

The present study was limited to a single clinical service. Such basic patient information such as demographics and clinical signs are available, but clinical details, antimicrobial treatment administered, the time of hospitalization period and clinical outcomes are often unavailable.

## 5. Conclusions

It would be necessary to trace through epidemiological studies the pathogens most commonly found in AV in order to better direct clinicians toward the optimal therapeutic choices. Furthermore, correct prophylaxis should be increased, even in the hospital. For developing countries, where the diagnosis of vaginal infections as well as clinical approaches are already difficult, it is necessary to develop products that are simple to apply, inexpensive and easy to handle. In the actual context of antibiotic resistance spread it is fundamental to administer correct antibiotics, especially accounting for the local epidemiological spread. Our work aims to provide an overview of the local diffusion of AV, the main players involved, paying attention to their resistance profiles in order to direct the clinician towards a more targeted therapeutic approach where possible. Since AV have implications for both pregnant women and unborn children, they need to be identified and managed in an optimal manner.

## Figures and Tables

**Table 1:** Positive, negative and total vaginal swabs analyzed during the five years of study, for each year and in total. The last column shows the relative percentage of incidence based on the five years summarized.

Samples (n.)	2015	2016	2017	2018	2019	2015–2019	Incidence %
Positive samples	117	286	275	234	264	1176	56.8%
Negative samples	118	188	203	163	221	893	43.2%
Total samples	235	474	478	397	485	2069	

**Table 2:** Number and percentage of positive incidence for microorganism growth samples by age group.

Age Group	% of Incidence
10–14	0.6%
15–24	7.2%
25–34	15.2%
35–44	17.9%
45–54	14.1%
55–64	26.5%
65–80	15.0%
81–95	3.4%

## Data Availability

Epidemiological data used to support the results of this study are included in the article.
